# Vitamin C, A and E supplementation decreases the expression of *HSPA1A* and *HSPB1* genes in the leukocytes of young polish figure skaters during a 10-day training camp

**DOI:** 10.1186/s12970-015-0069-8

**Published:** 2015-02-11

**Authors:** Małgorzata Żychowska, Zbigniew Jastrzębski, Grzegorz Chruściński, Monika Michałowska–Sawczyn, Alicja Nowak-Zaleska

**Affiliations:** Department of Health Promotion, Gdańsk University of Physical Education and Sport, Ul. Górskiego 1, 80-360 Gdańsk, Poland; Department of Physical Education, Gdańsk University of Physical Education and Sport, Ul. Górskiego 1, 80-336 Gdańsk, Poland

## Abstract

**Background:**

Overexpression of *HSPA1A* and *HSPB1* has been shown to indicate stress and the degradation of damaged proteins. Therefore, the expression of these genes is often evaluated during exercise. Vitamin supplementation in young athletes may affect the expression of these genes, and help to maintain health and improve the effects of training.

**Methods:**

Fourteen top junior female athletes (age 14–15y ± 0.3 SD, body mass 51 kg ± 5 SD, and BMI of 20.15 ± 0.9 SD, time in professional training 8.5 y ± 0.5 SD) attended a conditioning camp that included meals planned by a team dietitian. To examine the effects of vitamin supplementation on antioxidant status we supplemented the athletes with either vitamin A (16 ug/kg/day), vitamin C (8 mg/kg/day) and vitamin E (1 mg/kg/day) or an inert placebo. Blood samples were taken before and after (12 h post) the camp to assess the relative expression of HSPA1A and HSPB1 mRNA in leukocytes via quantitative reverse transcription polymerase chain reaction (qRT-PCR).

**Results:**

Overall, participants trained ~135 min daily (1345 min total). No statistically significant differences in *HSPA1A* and *HSPB1* expression were observed between the groups before the camp. In the unsupplemented group, there was a non-statistically significant increase in *HSPA1A* expression (100% change) and a significant increase (37% change, p < 0.05) in *HSPB1* expression over the study period. The supplemented group experienced a significant decrease in *HSPA1A* (40% change, p = 0.01) and *HSPB1* (25% change p = 0.03) expression over the study period.

**Conclusion:**

Our results indicate that supplementation with antioxidant vitamins decreases *HSPA1A* and *HSPB1* mRNA expression in leukocytes, and thereby may reduce exercise-induced stress in young athletes, not only during training, but also in sports competitions.

## Background

Heat shock proteins are well-known effectors of the cellular stress response. Transcription of the genes encoding for heat shock proteins can be induced by numerous factors, including thermal, oxidative and environmental stress, as well as exercise and pathophysiological processes [1-5]. High and moderate intensity endurance exercise alters gene expression in human white blood cells [6]. Changes in gene expression in the muscle are also reflected in peripheral blood leukocytes, and indicate a systemic response to exercise. Increased expression of *HSPA1A* and *HSPB1* prevents apoptosis and DNA damage [7], and overexpression of these genes enhances tolerance to thermal stress. Increased *HSPB1* expression is also associated with the degradation of damaged proteins [8,9]. Physical effort performed at elevated temperatures and the large amount of heat generated endogenously by the organism during exercise are both important factors that can stimulate the expression of these genes [10].

The anaerobic character of the physical exercise performed by figure skaters causes muscle damage and increased production of reactive oxygen species (ROS), leading to oxidative stress [11,12]. Supplementation with antioxidants may prevent oxidative stress [13,14] and muscle damage [15] during intensive training. The antioxidant vitamins C and E are commonly used as sports supplements [16]. Leukocytes, and in particular granulocytes, have an enormous capacity to produce ROS [17]. It has been suggested that leukocytes and erythrocytes in peripheral blood respond differently to supplementation, with leukocytes having a greater sensitivity than erythrocytes [18], and a reduction in oxidative stress occurs in response to supplementation with vitamins C and E [18]. Increased total antioxidant status (TAS) and higher levels of plasma iron have been shown in athletes treated with vitamin C and E after endurance training [19]. In addition to oxidative stress and environmental stress, temperature can also induce the expression of genes encoding for heat shock proteins. The greatest exercise-induced increase in expression of heat shock proteins was seen in HSP70 and HSP27, which are considered to be sensitive to exercise training [20].

In this study, we investigated whether supplementation with the antioxidant vitamins C, A, and E changes the expression of *HSPA1A* and *HSPB1* mRNA in young female skaters undergoing long-term physical exertion. We hypothesized that increasing their antioxidant capacity would attenuate the effects of exercise-induced stressors and thereby inhibit the expression of *HSPA1A* and *HSPB1* in the supplemented group. We assumed that uniform environmental conditions, similar diets, and comparable training intensities would allow us to properly assess the impact of antioxidant supplementation on expression of the *HSP* genes.

## Methods

### Characteristics of the study group

The study participants were 14 skaters from a top Polish skating club, i.e. junior figure skating soloists attending a 10-day training camp at the Central Sport Centre. Participants were 14–15 years old (mean 14.4 y ± 0.3 SD) with an average body weight of 51 ± 5 kg and height of 159 cm ± 4 SD. Their length of time in professional training was 8 years (mean 8.5 ± 0.5 SD). Figure skating in Poland is experiencing a crisis; in the 14–15 year old age group there are only 20 female trainees in this discipline. Most of them are in one club, Unia Oświęcim, which has a history of achieving high athletic performance in Poland and in the international arena, and continues to do so.

Investigating skaters at a training camp ensured a similar diet, circadian rhythm (similar bed times and wake up times), and training intensity. Based on their responses to our questionnaire, the respondents’ diets before the camp was poor in vitamins, including A, C and E. The athletes were sexually mature as noted in the interviews. In addition to the interview, the coach kept detailed records of the menstrual cycles of the skaters to take this into account during training. Our athletes menstruated regularly. Twelve of them were in the estrogenic phase and only two were in the progesterone phase (18th and 20th days of the cycle). Based on the date of their first menarche, we did not note any acceleration or deceleration in development.

The skaters were divided into two groups: one group received dietary supplementation with vitamins (A, C and E) and the other group received a placebo. The group assignments were based on individual competition rankings. The supplemented group included the athletes ranked 1, 3, 5, 7 etc. on the list, while the unsupplemented group included the athletes ranked 2, 4, 6, 8, etc. According to the guidelines of the Helsinki Declaration, participants in the experiment and their parents were informed in detail about the test procedure and provided their written consent for participation in the project. The study protocols received ethical approval from the Ethical Committee of the Regional Medical Chamber (KB-27/14).

### Training

**Figure 1:**
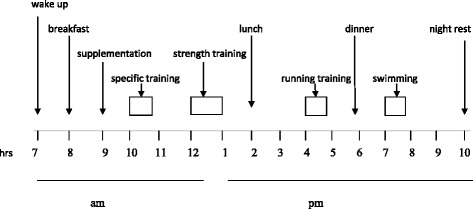
**Typical day organizations after the camp.**

The camp took place in the Olympic Training Center in June 2013. During the camp, the skaters were subjected to 1395 minutes of training, which was prepared and approved by their coach and involved different training zones determined on the basis of heart rate measured and recorded by a Sport Tester (Table 1). A total time of 10 minutes was designated for the phosphocreatine, 850 minutes for the lactate, and 535 minutes for the oxygen training zone. The average daily training lasted 125 minutes. A typical daily schedule and training load are presented in Figure 1 and in Table 1.

**Table 1:** **Ten days training load and specific type of physical exercises during the camp**

**Training loads performed by the subjects**							**Specific type of physical exercises**				
**Day of week**	**AP [min]**	**Mean HR**	**MAAP [min]**	**Mean HR**	**ANLP [min]**	**Mean HR**	**Day period**	Specific training* [min]	**Strength [min]**	**Running [min]**	Swimming** [min]
Tuesday	90	162(±5)	60	185(±10)	-	-	Morning	45	60		
							Evening			35	45
Wednesday	45	164(±10)	110	182(±8)	-	-	Morning	45	50		
							Evening			40	45
Thursday	45	160(±5)	90	179(±8)	5	190(±10)	Morning	45	60		
							Evening			35	45
Friday	40	167(±5)	110	180(±10)	-	-	Morning	45	50		
							Evening			40	45
Saturday	90	165(±7)	60	185(±7)	-	-	Morning	45	60		
							Evening			40	45
Sunday	Free day										
Monday	45	159(±5)	100	180(±6)	-	-	Morning	45	60		
							Evening			40	45
Tuesday	90	158(±5)	60	181(±5)	-	-	Morning	45	50		
							Evening			40	45
Wednesday	45	158(±10)	60	180(±5)	-	-	Morning	45	60		
							Evening			40	45
Thursday	45	158(±10)	95	186(±5)	5	192(±12)	Morning	45	50		
							Evening			35	45
Friday	-	-	105	182(±4)	-	-	Morning	45	60		
							Evening			40	45
Total Load [min]	535		850		10		Total Load [min]	450	560	385	450
Total	1395						Total	1395			

AP – Anaerobic Performance; MAAP – Mixed Aerobic-Anaerobic Performance; ANLP – Anaerobic Non Lactate Performance.

*Specific training - jumps on dry, lift and technical elements of figure skating.

**Exercises at the pool were treated as health wellness.

### Supplementation

None of the participating athletes was taking vitamin supplements at the start of the experiment. Exceptionally small doses of vitamins were taken for a short time in cases of sickness. Considering the age of athletes and the potential negative impacts of hypervitaminosis on health, vitamins were supplemented at the doses recommended for young people as follows: 16 μg/kg/day vitamin A, 8 mg/kg/day vitamin C, and 1 mg/kg/day vitamin E. These doses are in accordance with the Polish standards for this age group. Vitamins given to the skaters in the supplemented group were dissolved in orange juice and served once daily during the morning meal. The unsupplemented group received orange juice without added vitamins. The vitamin supplement was produced by TRIPOL pharmacy and was available in solution. The athletes received vitamin supplementation only during the camp.

### RNA extraction and quantitative real-time polymerase chain reaction (qRT–PCR)

Two ml of peripheral blood were collected from the ulnar vein of each participant at the beginning and end of the training camp. Prior to RNA extraction, erythrocytes were lysed and discarded using RBCL buffer (A&A Biotechnology, Poland). The leukocytes were lysed using Fenozol (A&A Biotechnology, Poland) and the RNA was subsequently precipitated by the method described by Chomczynski and Sacchi [21]. The extracted RNA was treated with DNaseI (Invitrogen) to digest any remaining DNA. cDNA was synthesized using the Transcript Me system (Blirt, Gdańsk, Poland) as per the manufacturer’s instructions. A mix of 10 μL master mix, 2 μL enzyme mix with M-MuLV reverse transcriptase and 2 μg RNA, and water, up to a final volume of 20 μL was added to each tube. Samples were incubated at 25°C for 10 min, 55°C for 30 min and 85°C for 5 min. qRT-PCR analyses for *HSPA1A* and *HSPB1* were performed using the Step One real-time PCR system (Applied Biosystems). Each qRT-PCR reaction mix consisted of 10 μl Semi Fast Sybr Green qPCR master mix (Biolone, UK), 2 μl of cDNA, 0.8 μL of each primer in 10 pM concentrate, in a final volume of 20 μL. Thermal cycling conditions included an initial hold at 95°C for 2 minutes, and than 40 cycles of 95 oC for 15 seconds, 60°C for 10 seconds and 72°C for 20 seconds. All samples were assayed in triplicate. Primer sequences are listed in Table 2. Target gene expression was normalized to the expression of the reference gene *Tata Box Protein* (*TBP*).

**Table 2:** **Sequence of primer used to PCR**

**Genes**	**Primer sequence:**
*HSPA1A*	Forward primer: ACTCCCGTTGTCCCAAGGCTTC
	Reverse primer: TCTGTCGGCTCCGCTCTGAGAT
*HSPB1*	Forward primer: AAGGATGGCGTGGTGGAGATCA
	Reverse primer: GAGGAAACTTGGGTGGGGTCCA
*TBP*	Forward primer: TGGACTGTTCTTCACTCTTGGC
	Revere primer: TTCGGAGAGTTCTGGGATTGTA

### Statistical analysis

Relative gene expression levels were calculated using the standard curve method. The normality of the distribution was checked with the Shapiro-Wilk test. For parametric analysis, the *t*-test for dependent samples (comparison of *HSPB1* expression before and after the camp) and the *t*-test for independent samples (comparisons between the supplemented and unsupplemented groups) were utilized. For non-parametric analyses, the Wilcoxon test (comparison of *HSPA1A* expression before and after the camp) and two-way ANOVA (comparison between groups for the two target genes) were used. Analysis of covariance (ANCOVA) was used to adjust for baseline values and to provide an unbiased estimate of the mean between-group differences (supplemented vs unsupplemented) in the expression of *HSPA1A* and *HSPB1*. All analyses were performed in Graph Pad Prism 6.0 (www.graphpad.com), except for the two-way ANOVA way and ANCOVA, which were done using STATISTICA version 10 (www.statsoft.com) Differences were considered statistically significant if p ≤ 0.05.

## Results

**Figure 2:**
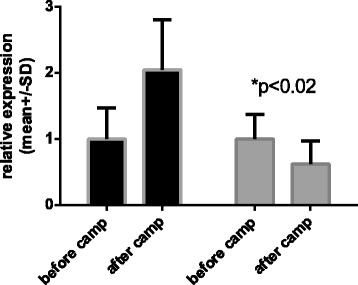
**Relative expression of** ***HSPA1A*** **normalized to** ***Tata Box Protein - TBP*** **(dark bars – unsupplemented group, gray bars supplemented group).** 1- before camp, 2- after camp.

**Figure 3:**
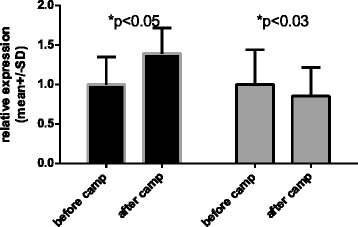
**Relative expression of the** ***HSPB1*** **gene normalized to** ***TBP*** **(dark bars – unsupplemented group, gray bars – supplemented group).** 1- before camp, 2- after camp).

Relative *HSPA1A* and *HSPB1* mRNA expression in the supplemented and unsupplemented groups are presented in Figures 2 and 3. In the unsupplemented group, there was a non-significant, 100% change in the expression of *HSPA1A* mRNA from before the camp to after the camp. Conversely, there was an approximately 38% statistically significant (p < 0.02) reduction in the relative expression of *HSPA1A* mRNA in the supplemented group over the study period (Figure 2). There were no significant between-group differences in the basal expression of the target genes before the camp.

Figure 2 shows the data of expression *HSPA1A* m-RNA involving the unsupplemented and supplemented groups, measured before and after the camp. To allow better visualization of the changes in relative expression, the pre-camp expression levels of both groups were set to 1.

**Figure 4:**
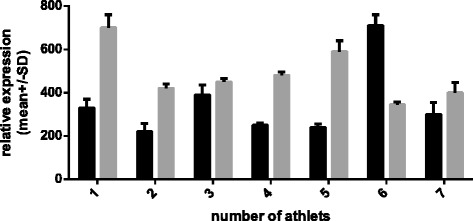
**Individual changes in** ***HSPA1A/TBP*** **expression in not supplemented group measured before (dark bars) and after (gray bars) the camp.**

**Figure 5:**
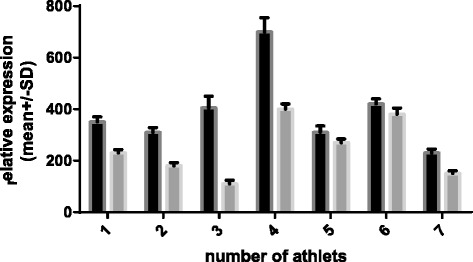
**Individual changes in** ***HSPA1A/TBP*** **expression in supplemented group measured before (dark bars) and after (gray bars) the camp.**

**Figure 6:**
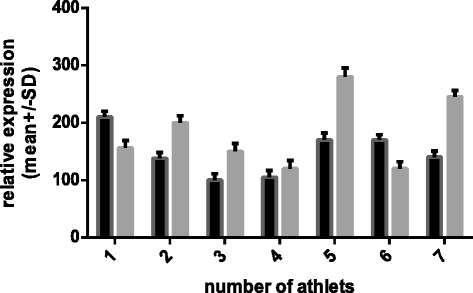
**Individual changes in** ***HSPB1/TBP*** **expression in not supplemented group measured before (dark bars) and after (gray bars) the camp.**

**Figure 7:**
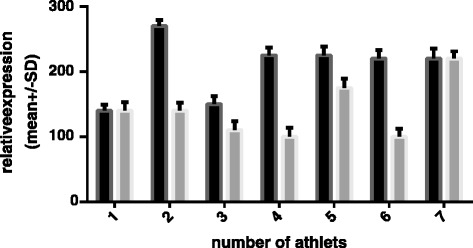
**Individual changes in** ***HSPB1/TBP*** **expression in supplemented group measured before (dark bars) and after (gray bars) the camp.**

The mean data of *HSPB1* m-RNA involving the unsupplemented group and the supplemented group are presented in Figure 3. To allow better visualization of changes in relative expression, the pre-camp expression levels of both groups were set to 1. There was a 0.38-fold increase in *HSPB1* in the unsupplemented group (p = 0.043) and a 0.16-fold decrease in the supplemented group (p = 0.023). However, there were no significant between-group differences in the basal expression of the target genes before the camp. There were between-group differences in the relative expression levels of both *HSPA1A* and *HSPB1* mRNA after the camp; however, the differences in *HSPA1A* mRNA expression were more prominent. Supplementation also changed the expression of the *HSPA1A* chaperone, *HSPB1,* relative to basal levels. We assume that the training camp eliminates the potential confounding influences of factors such as dietary differences, additional personal activities besides training, and other environmental conditions on the gene expression. Of course, personal sensitivity to exercise and supplementation can vary and equivalent environmental conditions cannot control for intrinsic differences in the expression of the target genes between individuals. For this reason, we investigated the relative changes in gene expression levels. The expression levels of *HSPA1A* were more variable among individuals than were the expression levels of *HSPB1* (Figures 4, 5, 6 and 7).

When the individual data for *HSPA1A* mRNA expression are considered, four athletes in the unsupplemented group had a two-fold increase in expression over the study period. Two other skaters showed less prominent changes, but their expression levels were also elevated. Only one of the skaters in the unsupplemented group had a decrease in the relative expression of *HSPA1A* mRNA (by approximately 50%).

Among the skaters receiving supplementation, the relative expression of the *HSPA1A* transcript decreased in all seven athletes. However, the magnitude of the change varied between individuals.

In general, the direction of the changes in relative gene expression were in line with the average results for the whole group. The unsupplemented athletes, with the exception of one female athlete, showed increased *HSPB1* expression to pre-camp levels (Figure 6). This increase was not dependent on the initial level of expression, and showed inter-individual differences. Relative expression of *HSPB1* decreased in all of the supplemented athletes; however, this change was less prominent than the change in *HSPA1A* expression (Figure 7).

Two-way ANOVA revealed a statistically significant group x time interaction for both target genes (Table 3). Analysis of covariance (ANCOVA), after considering the basal (pre-camp) value showed a significant difference between the groups in HSPA1A expression (p < 0.01) and no significant between-group difference in *HSPB1* expression (p = 0.17).

**Table 3:** **Results ANOVA two – way and power of analysis**

	**Supplemented group**	**Un-supplemented group**		**ANOVA 2-way**				**ANCOVA**
**gene**	**PRE**	**POST**	**PRE**	**POST**	**Differences group x time**	**Effect size(ES)**	**Power**	**covariance**
	Normalized expression (Mean ± SD)							
*HSPA1A*	389.3 ± 151.2	245.7 ± 111.5	348.6 ± 169.8	483.6 ± 122.2	≤0.03	0.37	0.68	p ≤ 0.04
*HSPB1*	207.1 ± 45.99	140.7 ± 44.20	147.6 ± 39.00	181.6 ± 62.30	≤0.05	0.52	0.91	p ≤ 0.03

## Discussion

There are many reports in the literature on the influence of oxidative stress during physical activity on the development of the inflammatory response and the resulting induction of HSP synthesis [5,18,19]. Under conditions of physical effort, the overexpression of genes encoding for HSP helps cells survive by protecting them from apoptosis. Morton et al. [22] claimed that the overexpression of HSP genes occurs after crossing a critical threshold, which is determined by individual difference in HSP expression propensity, training level, and also ROS levels. Evidence suggests that the level of HSP gene expression may indicate stress on the organism [5,18,19]; however, it is difficult to interpret changes in HSP gene expression. An increase in expression may indicate a high level of exposure to a stressor and that the critical threshold has been crossed. However, a decrease in the expression of these genes may reduce the organism’s ability to tolerate stress [22].

Lui et al. [5], Tauler et al. [18] and Aguilo et al. [19] have all reported that an increase in antioxidant status will result in a decrease in oxidative stress and, consequently, the applied physical effort will result in lower stress. In this study, we used the group setting of an intense skating training camp to eliminate variations in diet, circadian rhythm, and additional, uncontrolled physical activity.

It is difficult to compare our results to previously published data since there have been few reports on this topic. The studies that have been performed used different experimental conditions, such as the nature of the physical exercise [23-26], the gender of the participants [4,27], the level of commitment to the sport (recreational or professional), various supplementations [18], and diverse tissues as the source of RNA. In this study, we examined the expression of *HSPA1A* and *HSPB1* in the leukocytes of junior female figure skaters. To our knowledge, there are no similar data available in the literature. The response to physical exercise is not only local but systemic, making analysis of relative gene expression in peripheral blood leukocytes a reasonable indicator of the whole body response to our experimental intervention [28]. Therefore, we hypothesized that antioxidant vitamins would influence the expression in leukocytes of genes which are known to be induced by oxidative stress [2,3]. Aguilo et al. [19] showed the influence of vitamin A, E, and beta carotene supplementation in a group of 18 male amateur athletes who were randomly assigned to groups. They found that physical exercise decreased the antioxidative status of the unsupplemented group only. In another study involving a group of amateur athletes, antioxidant supplementation for three months prior to high intensity training was shown to increase the antioxidative status of their leukocytes [18].

In contrast, Taghiyar et al. [29] examined vitamin C and E supplementation in a group of women practicing aerobics and found a correlation between supplementation and decreased levels of markers of muscle damage. Interestingly, decreases in the baseline expression levels of *HSPA1A* have been seen as a training adaptation in highly qualified athletes in comparison to a control group (data not published). Due to the small amount of genetic research done on professional athletes, it is difficult to choose a suitable methodology for studies such as this one.

More data exist regarding changes in *HSPA1A* expression after physical exercise than exist for *HSPB1*, but they remain ambiguous. The results vary between studies since changes in *HSPA1A* expression are dependent on the type of effort and the type of muscle fiber involved in the particular exercise [25,26]. Thus, Ryan et al. [30], in a study involving young men subjected to two hours of effort on a treadmill under heat stress conditions, noted only minor changes in *HSPA1A* expression in peripheral blood leukocytes. On the other hand, Lui et al. [5] examined a group of rowers and observed elevated expression of *HSPA1A* even four weeks after the training period. Therefore, it seems reasonable to examine athletes during their rest phase, as well as during physical training. Despite great interest in heat shock proteins, it seems that only some of the stress factors that can induce expression of HSP encoding genes (such as physical effort) are currently known [31].

Our research involved a group of young junior figure skaters and there are no published, comparable data from this particular discipline. Additionally, the tested group had never undergone supplementation. Besides the type of sport, the gender of the participants may also influence the results. Since hormones can have a huge impact on gene expression, comparing females and males is a difficult task. Chang et al. [27] supplemented women with estrogen for three days and did not observe any changes in the level of HSP70 protein in leukocytes; however, this does not exclude between-gender differences. We expect that the response to supplementation of athletes who are in the same estrogenic phase should be similar. However, it is difficult to discuss our results in this regard due to the lack of comparable studies in the literature. We recognize that the potential for extrapolation of our results, which are based on the responses of a specific group of 14 people, to the general population is limited. However, supplementation with antioxidant vitamins significantly reduced the expression of *HSPA1A* and *HSPB1* genes in our study participants, which might indirectly indicate an increased antioxidant capacity of their leukocytes and, therefore, a lower need for the expression of genes involved in the apoptotic pathway and the degradation of damaged proteins.

## Conclusion

Our results indicate that supplementation with antioxidant vitamins such A, C and E significantly decreases the expression of *HSPA1A* and *HSPB1* in the leukocytes of young soloist figure skaters subjected to heavy training load. It is likely that our results were affected by the vitamin-deficient status of the athletes at the start of the study; growing young women involved in high intensity training should be consuming much higher levels of vitamins than these women were.

## Abbreviations

ROS: Reactive oxygen species
TAS: Total antioxidant status
*TBP*: Tata box protein gene
*HSPA1A*: Gene coding HSP70 kDa protein
*HSPB1*: Gene coding HSP27 kDa protein
